# *Lactobacillus rhamnosus* GG and Biochemical Agents Enrich the Shelf Life of Fresh-Cut Bell Pepper (*Capsicum annuum* L. var. grossum (L.) Sendt)

**DOI:** 10.3390/foods9091252

**Published:** 2020-09-07

**Authors:** Kandasamy Saravanakumar, Anbazhagan Sathiyaseelan, Arokia Vijaya Anand Mariadoss, Ramachandran Chelliah, Xiaowen Hu, Deog Hwan Oh, Myeong-Hyeon Wang

**Affiliations:** 1 Department of Medical Biotechnology, College of Biomedical Sciences, Kangwon National University, Chuncheon 24341, Korea or saravana732@gmail.com (K.S.); sathiyaseelan.bio@gmail.com (A.S.); mavijaibt@gmail.com (A.V.A.M.); huxiaowen9520@gmail.com (X.H.); 2 Department of Food Science and Biotechnology College of Biotechnology and Bioscience, Kangwon National University, Chuncheon 24341, Korea; ramachandran865@gmail.com (R.C.); deoghwa@kangwon.ac.kr (D.H.O.)

**Keywords:** probiotics, *Lactobacillus rhamnosus*, bell pepper, food quality, foodborne

## Abstract

This work analyzed the individual and combined effects of biochemical additives and probiotic strain *Lactobacillus rhamnosus* GG on red and yellow fresh-cut bell pepper (R- and Y-FCBP, respectively) stored at two different temperatures (4 °C and 15 °C) for 15 days. The results revealed that the combined application of biochemical additives and *L. rhamnosus* GG inhibited the colonization of total bacterial counts (25.10%), total *Salmonella* counts (38.32%), total *Listeria* counts (23.75%), and total fungal counts (61.90%) in FCBP. Total bacterial colonization was found to be higher in R-FCBP (1188.09 ± 9.25 CFU g^−1^) than Y-FCBP (863.96 ± 7.21 CFU g^−1^). The storage at 4 °C was prevented 35.38% of microbial colonization in FCBP. Importantly, the *L. rhamnosus* GG count remained for up to 12 days. Moreover, the combined inoculation of the biochemical additives and *L. rhamnosus* GG treatments (T3) maintained the quality of R- and Y-FCBP for up to 12 days at 4 °C without any loss of antioxidant properties. This work reports the successful utilization of *L. rhamnosus* GG as a preservative agent for maintaining the quality of FCBP by preventing microbial colonization.

## 1. Introduction

Bell pepper (in other words, paprika or sweet pepper) is one of the leading greenhouse products in South Korea. Annually, about 20,000 tons of bell pepper is produced and exported to various countries. In particular, 60% of Korean bell pepper are commercialized in Japanese markets [1,2]. However, the transportation of bell pepper in the form of fresh-cut products (FCP) is more voluminous in terms of kilograms than that of whole bell pepper, using the same container, and this can increase the economic value and reduce the transportation charges. Moreover, people are very interested in consuming the fresh-cut form of the sweet pepper without loss of nutritional value. However, there is a high risk of food pathogen contamination during the preparation of fresh-cut bell pepper (FCBP) by cutting, peeling, and slicing methods, which lead to tissue damage, the release of nutrients, and attraction of microbial contamination, resulting in the loss of flavor and texture, as well as browning [3]. Moreover, the consumption of improperly processed FCP may lead the severe illness to human. A list of food pathogen (*Salmonella*, *Escherichia coli*, or *Listeria monocytogenes*)-based outbreaks have been reported in past decades, such as the infection of listeriosis by *L. monocytogenes* due to consumption of various fresh fruits and vegetables [4] in 1994, *E. coli* (O11:H7) infection in the United States [5], and Shiga toxin-producing *E. coli* O104 causing hemolytic–uremic syndrome in Europe in 2014 [6]. Moreover, *L. monocytogenes* are associated with 20–30% of the fatalities, which is higher than that of other foodborne pathogens [7], and its contaminations in raw foods and lettuce have been reported from the China and Korea, respectively, which caused the listeriosis [8,9]. The *E. coli* O157: H7 and *Listeria* spp are known to significantly contaminate several fresh products of lettuce, cucumber, carrot, soybean sprouts, packed fresh-cut salad, apples, melon, and peaches [9,10,11,12,13,14]. The Korea Food and Drug administration (KFDA) has provided prevention measures to control the foodborne pathogenic (*Salmonella* sp., *Listeria* sp., *E. coli*, and fungi, etc.) infections [15]. Also, another report has consolidated the global foodborne outbakes related to the FCP and key prevention measures [16].

To inhibit microbial contamination in FCP, several processing methods are currently applied, including low-temperature refrigeration, minimal processing, sanitization solution washing (hydrogen peroxide, chlorine, ozone, and organic acids), anti-browning agents, chemical dipping in edible coating, and antibacterial essential oil treatments [17,18,19,20]. However, the effect of these approaches are different based on the response of the FCP; therefore, it is important to optimize appropriate conditions to expand the shelf life of FCP based on the fruits and vegetables [19]. Therefore, a novel invention is needed for hygienic food processing and storage to avoid food safety-related issues and maintain the good quality of FCP for better transportation and direct commercialization [3]. However, among various sanitizer-based treatments, the biopreservation techniques using living probiotic lactic acid bacteria (LAB) are promising as far as having an inhibitory effect on foodborne pathogens through the production of various metabolites, such as lactic acids and peptides [21,22]. Also, probiotics are advantageous to the host by improving intestinal microbiota and immunity [22]. Some of the probiotics are known to be utilized as preservative agents for FCP, and particularly *L. rhamnosus* GG is reportedly expanding the shelf life of FCP, such as pineapple, apple, pears, and melon [23,24,25,26]. However, there is no work reporting the utilization of *L. rhamnosus* GG to expand the shelf life of FCBP. Therefore, the present work tested the effect of *L. rhamnosus* GG combined with biochemical additives on the shelf life of FCBP.

## 2. Materials and Methods

### 2.1. Bell Pepper and Microbes

Two different colors (red and yellow) of fresh and healthy bell pepper used in this study were purchased from local farmers in Chuncheon, Republic of Korea, and transported to the laboratory. The bacterial strains used were *L. monocytogenes* (ATCC 19118), *S. Typhimurium* (ATCC14028), and *L. rhamnosus* GG (ATCC 53103) from the American type of the culture collection located in University Blvd, Manassas, VA, United States. The culture media used were tryptic soy broth (TSB) and tryptic soy agar (TSA); xylose–lysine–desoxycholate agar (XLD); Polymyxin acriflavine lithium chloride ceftazidime aesculin mannitol (PALCAM) agar; and de Man, Rogosa, and Sharpe agar and broth (MRSA and MRSB, respectively) from Oxoid (Basingstoke, Hampshire, UK). Sterile peptone water was purchased from BactoTM, Difco, MD, United States. Phosphate-buffered saline (PBS; Corning Mediatech, Inc., Corning, NY, USA), sodium benzoate, ascorbic acid, and DL-α-tocopherol acetate were obtained from Sigma Aldrich, the Republic of Korea.

### 2.2. Preparation of Biochemical Additive Solution and Bacterial Inoculum

The biochemical additive solution was prepared by dissolving the 20 g L^−1^ of ascorbic acid, 200 mg. L^−1^ of sodium benzoate, and 2 g L^−1^ of DL-α-tocopherol acetate in distilled water. The bacterial strains, such as *L. monocytogenes* and *S. Typhimurium*, were retrieved from 20% glycerol stock by inoculating on TSA media by a simple streak plate method, and were allowed to grow in an incubator at 37 °C for 24 h. The single colonies of bacteria were transferred to 10 mL of TSB and incubated for 15–18 h at 37 °C. Then the cells were collected by centrifugation at 3000 rpm for 10 min. The harvested cells were resuspended in sterile peptone water and the concentration of the cells was adjusted to 10^5^ CFU mL^−1^ for *L. monocytogenes* and *S. Typhimurium*, and then mixed in 10 mL of peptone water to obtain the foodborne cocktails (FBC). The probiotic strain *L. rhamnosus* GG was retrieved from the stock, according to instructions provided from ATCC, and cultured in MRSA. The cells were grown in MRSB until the late stationary phase in an incubator at 37 °C. Afterward, the cells were collected by centrifugation at 9000 rpm for 10 min at 8 °C, and the concentration of the cells was adjusted to 10^8^ CFU mL^−1^ for *L. rhamnosus* GG. The final concentration of each cells were checked by culturing appropriate dilution XLD for *Salmonella*, Palcam agar for *Listeria*, and MRSA for *L. rhamnosus* GG.

### 2.3. Preparation of the FCBP and Treatments

**Scheme 1:**
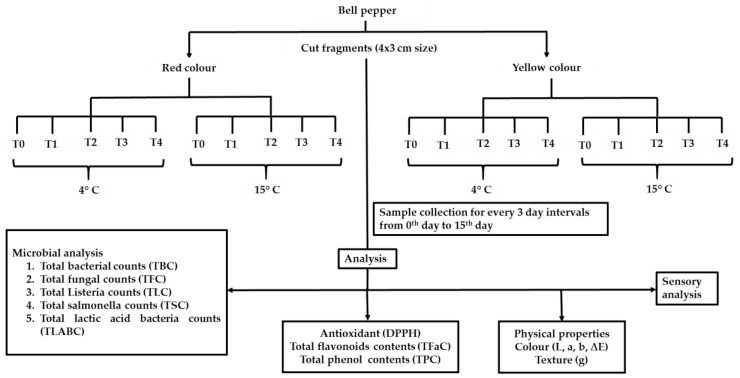
Schematic representation of the experimental design.

To apply various treatments, the yellow and red colors of bell pepper were cut into fragments sized at 4 × 3 cm using a sterilized knife, and air-dried and divided into a total of 10 groups for each color of FCBP. This was done in triplicates, in which five groups were at 4 °C and five groups were at 15 °C preservation. Each group of FCBP was treated (1:3 *w*/*v*) for 2 min at 120 rpm in any one of the following treatments: T0 = untreated, T1 = biochemical additives solution-treated, T2 = biochemical additive solution with FBC, T3 = biochemical additive solution with probiotic strain *L. rhamnosus* GG (10^8^ CFU mL^−1^), T4 = biochemical additive solution with FBC and *L. rhamnosus* GG (10^8^ CFU mL^−1^). After the treatments, FCBP samples were aseptically dried by laying on sterile blotting paper in a clean cabinet to remove the water vapors. Then the samples were packed in a sterile polypropylene container with a size of 9.5 × 7.5 cm and stored in two different temperatures (4 °C and 15 °C) and 70% relative humidity. The microbial quality, nutritional, color, and sensory properties of FCBP were frequently measured by collecting the aliquots of samples at three-day intervals from 0 to 15 days. The detailed experimental design was presented in Scheme 1. The concentration of the sodium benzoate in the FCBP was determined by using the methods described earlier [27].

### 2.4. Enumeration of Microbial Colonization

To enumerate the microbial colonization, 5 g of the FCBP were dissolved in 10 mL of the sterile distilled H_2_O and serially diluted up to 10^−7^. The 50 µL of 10^−5^ dilution was spread on the nutrient agar (NA) for total bacterial counts, XLD for total *salmonella*, PALCAM for total *Listeria*, MRSA for total LAB, and potato dextrose agar (PDA) for total fungal enumeration [26,28]. The plates with MRSA, XLD, PALCAM agar, and NA were incubated at 37 °C for 24 h, while the PDA plates were incubated at 28 °C for four days. After the incubation period, the microbial counts were enumerated using the standard methods, as described [28]. The colonies corresponding to the *Listeria* sp. were confirmed based on the colony morphologies, as indicated with a grey–green color with a black center and a black halo. Similarly, the *salmonella* sp. were identified based on the red colonies with black centers for some colonies.

### 2.5. DPPH Scavenging, Total Phenol, Flavonoids, Color, Texture, and Sensory Properties

A total of 70 g of the FCBP was collected from each treatment every three days (0, 3, 6, 9, 12, 15 days) and subjected to water extraction. This water extract was used for 2,2-diphenyl-1-picrylhydrazyl (DPPH) scavenging, total phenol, and total flavonoids assays. The DPPH assay was performed according to the methods reported earlier [29], and the Folin–Ciocalteu assay was adapted to measure the total phenol content (TPC) [30]. The hardness (g) was measured by texture analyzer (Brookfield, AMETEK GmbH, Lorch, Germany). The color changes, including *L* (lightness), *a* (redness), and *b* (yellowness) were observed by colorimeter (CR300; Minolta Co., Osaka Japan). Sensory properties of the FCBP were monitored by nine trained experts, and the sensory quality was scaled according to the method described earlier, with minor modifications [31]. The sensory quality was scored from 1 to 10, with 1–2 meaning extremely poor, 3–4 as poor (limit edibility), 5–6 as acceptable (limit of marketing), 7–8 as looks fresh and good, and 9–10 as excellent quality.

## 3. Results and Discussion

### 3.1. Microbial Changes

There is so far no report on the utilization of probiotic bacteria in combination with a biochemical additive solution to improve the quality of FCBP through inhibiting foodborne microbes. Therefore, this work evaluated the individual and combined effects of biochemical additives and *L. rhamnosus* GG on the quality and shelf life of FCBP. The application of probiotics in FCP was found to be advantageous in inhibiting foodborne pathogens. The descriptive statistical analysis of the microbial colonization in R- and Y-FCBP concerning the various time intervals, treatments, and storage temperatures is presented as supplementary tables (SI; Tables S1 and S2). In the present work, microbial count in FCBP was significantly different between the treatments, color of FCBP, temperature, and intervals (*p* < 0.05; Table 1). The total bacteria count (TBC) ranged from 412.2 ± 2.89 to 1959.30 ± 6.14 CFU g^−1^ for the treatments, and it was found to be higher in T0 and lower in T3. In terms of the color of FCBP, the maximum was found in R-FCBP (1188.09 ± 9.25 CFU g^−1^) and the minimum in Y-FCBP (863.96 ± 7.21 CFU g^−1^). In the case of preservation temperatures, TBC was observed more at 15 °C than at 4 °C. Furthermore, the colonization of TBC remarkably increased with increasing days of preservation. Thus, *L. rhamnosus* GG was highly efficient in inhibiting bacterial colonization in FCBP. The consumption of probiotic-treated fruits have been reported to reduce the incidence of cancer and cardiovascular diseases [32,33,34]. Lactic acid bacteria are known to potentially prevent bacterial colonization, due to their production of health-promoting metabolites [35].

The total *Salmonella* count (TSC) was observed to be higher in T2 (640.43 ± 2.34 CFU g^−1^) and lower in T1 and T3. TSC colonization was recorded more in R-FCBP (225.56 ± 4.25 CFU g^−1^) than in Y-FCBP (129.52 ± 2.15 CFU g^−1^). It was found higher with the preservation temperature of 15 °C (239.16 ± 8.14 CFU g^−1^) and lower in 4 °C (115.98 ± 6.15 CFU g^−1^). The TSC was found higher in the 0 day interval, but it decreased by days 3 and 6, followed by an increase in the 15 day interval. The combined application of biochemical additives and *L. rhamnosus* GG controlled the colonization of *Salmonella* in FCBP. TLC increased in T2 (431.36 ± 6.11 CFU g^−1^) and decreased in T1 and T3. The population of TLC was found to be greater in R-FCBP (149.69 ± 1.96 CFU g^−1^) and lower in Y-FCBP (64.47 ± 2.89 CFU g^−1^). It increased at the preservation temperature of 15 °C (125.04 ± 1.69 CFU g^−1^) and decreased at 4 °C (89.24 ± 2.13 CFU g^−1^). The co-inoculation of *L. rhamnosus* GG significantly inhibited the growth behavior of *Listeria* more than that of *Salmonella* in FCBP. The present work observed a negative correlation between TSC and total lactic acid bateria count (TLABC) (Table 1). Similarly, earlier work has reported that *L. rhamnosus* GG co-inoculation successfully controls the growth of *L. monocytogenes* better than that of *Salmonella* in apple wedges [22]. Several studies have reported the broad spectrum of antagonistic activity of *L. rhamnosus* GG against foodborne pathogens [36].

The TFC population was higher in T0 (1000.87 ± 4.55 CFU g^−1^) and lower in T3 (115.57 ± 5.79 CFU g^−1^). The population of TFC was found to be greater in R-FCBP (932.94 ± 3.93 CFU g^−1^) and smaller in Y-FCBP (397.55 ± 3.71 CFU g^−1^). It was also greater at the storage temperature of 15 °C (1131.43 ± 2.30 CFU g^−1^) and lower at 4 °C (199.06 ± 9.29 CFU g^−1^), and increased with increasing time of storage. The biochemical additives and low molecular weight molecules from viable cells of *L. rhamnosus* GG might be involved in the inhibition of fungal colonization in FCBP. Similarly, the *Lactobacilli* are known to inhibit fungi in dairy products [37,38]. The *Lactobacilli* (LAB) was absent in T0, T1, and T2, but present in T3 and T4, because LAB was not treated in T0, T1, and T2. Moreover, the *L. rhamnosus* GG count was remained for up to 12 days in T3 and T4 at both temperatures. The presence of the LAB significantly reduced the colonization of foodborne pathogens, such as *Salmonella*, *Listeria*, total bacteria, and total fungi. The results indicate that the biochemical additive solution combination with viable cells of *L. rhamnosus* GG treatments prevented foodborne pathogenic colonization in both red and yellow FCBP.

### 3.2. Phytochemical Changes and Antioxidant Properties

The results of antioxidant and phytocomponents, such as total flavonoids and total phenol, in R- or Y-FCBP treated with biochemical additives and *L. rhamnosus* GG and stored at two temperatures for 15 days are presented in Table 2. The results reveal that antioxidants and phytocomponents were significantly different between the treatments, type of FCBP, storage temperatures, and periods (*p* < 0.01). The antioxidant activity in terms of the DPPH scavenging was found to be higher with FCBP treated with T4 (biochemical additives and *L. rhamnosus* GG), while it was found to be lower at T0 (biochemical additives-alone treated) and T2 (biochemical additives and FBC-treated), due to the loss of natural compounds in FCBP through colonization of the foodborne pathogens. Also, the present results evidenced the correlation between the total flavonoids and phenol in the FCBP. Similarly, a previous study has reported that the application of probiotic lactic acid bacteria does not affect the quality of fresh-cut melon, and maintains the total phenol and antioxidants [31]. The biochemical additives maintained the quality of FCBP, but the effect was not better than that of *L. rhamnosus* GG. Since the usage of a higher dosage of sodium benzoate as the preservative agent is dangerous to human consumption, the present study used the lower concentration of the sodium benzoate as one constituent in the biochemical additive solution. To ensure its safety in human consumption, the concentration of sodium benzoate in the FCBP treated with the biochemical additive solution were determined, and it was found to be <0.001%, which is safe for the human consumption, according to “generally recognized as safe” (GRAS) compounds by the United States Food and Drug Administration [39].

### 3.3. Color and Texture Sensory Changes

**Figure 1:**
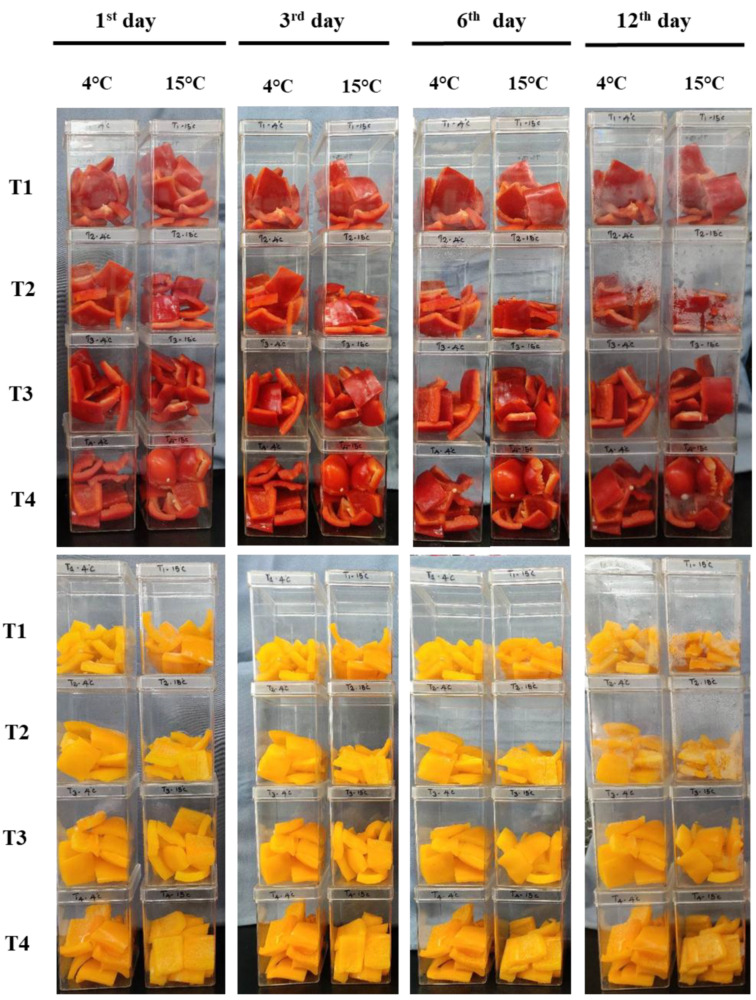
Effect of the *Lactobacillus rhamnosus* GG and biochemical additive treatments on visual appearance and sensory properties of red (R) and yellow (Y) fresh cut bell pepper (FCBP) in relation to day 1, day 4, day 6, and day 12 intervals.

Table 3 shows physical–visual changes of FCBP treated with biochemical additives or *L. rhamnosus* GG and stored at two different temperatures (4 °C and 15 °C) for 15 days. In general, the analysis of physical and visual parameters, including *L* (lightness), *a* (redness), *b* (yellowness), and texture is essential to understand the visual status of the fresh-cut produce. The results indicate that the color parameters, such as *L*, *a*, *b*, and texture significantly varied between the treatments, storage temperature, and days of storage (*p* < 0.01), and the color change (∆*E*) showed significant variation between treatments and days of preservation, but not between storage temperatures (*p* < 0.05). After 12 days of intervals, it was not possible to analyze physical and visual parameters, due to the deterioration of the FCBP. The color parameter *L* decreased with the increase of storage periods. The present results indicate that the storage temperature did not affect the browning of FCBP. The inoculation of pathogens’ FBCs with biochemical additives did not affect the color properties of FCBP [38,40]. However, it is difficult to determine the browning of bell pepper based on the color parameters alone, because it naturally has multiple colors, including green, yellow, orange, red, etc. However, the treatment of the biochemical additives or *L. rhamnosus* GG in FCBP challenging with FBC did not cause the browning, due to the prevention of the foodborne pathogenic colonization (Figure 1). Also, the biochemical additive agents used are reportedly preventing the browning of fruits, including fresh-cut red apples [40,41,42], pomegranate [43], and fresh-cut jackfruit [44]. Moreover, the texture of the FCBP is maintained by probiotic treatments. Similarly, the application of *L. rhamnosus* GG does not affect the quality of apple [25], pineapples [45], and cantaloupe [31].

**Figure 2:**
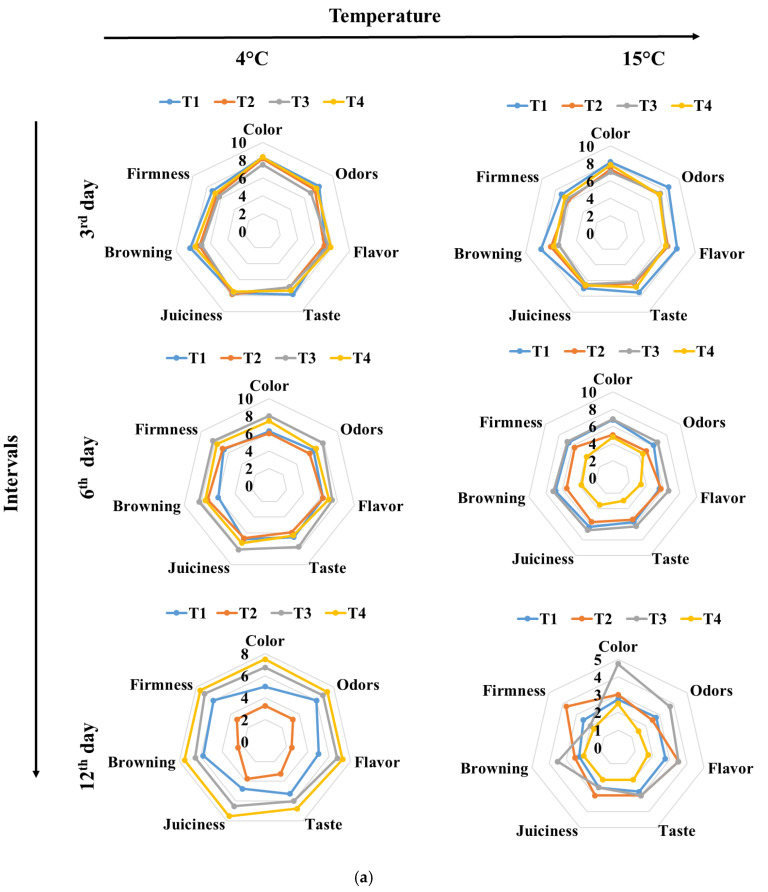

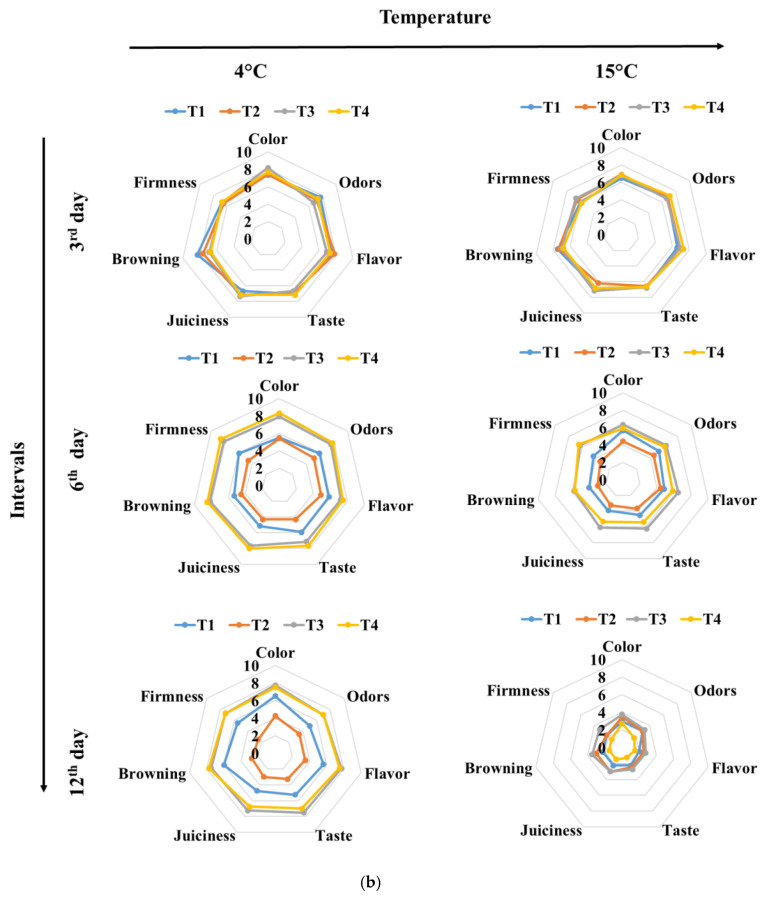
(**a**) Effect of the *Lactobacillus rhamnosus* GG and biochemical additives treatments on the sensory properties of red (R) fresh-cut bell pepper (FCBP) in comparison to day 4, day 6, and day 12 intervals. (**b**) Effect of the *Lactobacillus rhamnosus* GG and biochemical agent’s treatments on the sensory properties of yellow (Y) fresh cut bell pepper (FCBP) in comparison to day 4, day 6, and day 12 intervals.

Sensory evaluation is one of the essential measures to ensure the quality of preserved fresh-cut vegetables and fruits. The present work evaluates the effect of individual and combined effects of biochemical additives and *L. rhamnosus* GG treatments on FCBP stored at two different temperatures for 15 days. FCBP was testable for up to 12 days, but later it was deteriorated through colonization of microbial and foodborne contaminants. Therefore, the biochemical additive and *L. rhamnosus* GG-treated FCBP stored at 4 °C and 15 °C after the 12th day were subjected to the sensory evaluation using an expert panel. The quality of FCBP was very poor when stored at 15 °C for 12 days, while the 4 °C storage showed promising sensory quality with limited commercialization (Figure 2a,b). The treatment of the additive solution did not show the significance at the fourth day interval, but later it prevented the colonization of the foodborne pathogens and maintained the quality for up to 8 days (T1). Inoculation with FBC and a biochemical additive solution (T2) was not effective at 15 °C for the storage of FCBP. However, the combined inoculation of the biochemical additives and *L. rhamnosus* GG treatments (T4 and T3) maintained the quality of red and yellow FCBP for up to 12 days at 4 °C of storage temperature without loss of nutritional values (Figure 2a,b).

## 4. Conclusions

This work assessed the combined and individual effects of biochemical additives and *L. rhamnosus* GG treatments on the preservation of FCBP at two different temperatures for 15 days. The results revealed that the application of the biochemical additive alone was effective until 8 days of the storage at 4 °C, while the combined treatment (T4) and *L. rhamnosus* GG alone (T3) treatments maintained the quality of the FCBP for 12 days at 4 °C with potential antioxidant properties by preventing the colonization of foodborne pathogens. Thus, the application of the probiotic strain improves the shelf life and quality of FCBP.

## Figures and Tables

**Table 1:** Determination of microbial population changes in red (R) and yellow (Y) fresh-cut bell pepper (FCBP) treated or untreated with *L. rhamnosus* GG and biochemical agents at different time intervals (days) and preserved in two different temperatures (4 and 15 °C). The results presented the estimated marginal means ± standard error (SE). ** Significant (*p* < 0.01); NS: not significant, TBC: total bacteria count, TSC: total *Salmonella* count, TLC: total *Listeria* count, TFC: total fungal count, TLABC: total lactic acid bacterial count, *F* = variation between sample means/variation within the samples.

Sources	Microbial Counts (CFU g^−1^)				
	TBC	TSC	TLC	TFC	TLABC
Treatments					
T0	1959.30 ± 6.14	1.83 ± 0.23	1.57 ± 0.05	1000.87 ± 4.55	0
T1	911.6 ± 2.56	0	0	753.61 ± 6.23	0
T2	1031.21 ± 8.74	640.43 ± 2.34	431.36 ± 6.11	836.28 ± 2.56	0
T3	412.2 ± 2.89	0	0	115.57 ± 5.79	86.30 ± 3.55
T4	491.87 ± 7.25	245.43 ± 5.26	102.48 ± 2.13	619.59 ± 6.13	83.14 ± 0.84
Colors					
R-FCBP	1188.09 ± 9.25	225.56 ± 4.25	149.69 ± 1.96	932.94 ± 3.93	33.79 ± 2.84
Y-FCBP	863.96 ± 7.21	129.52 ± 2.15	64.47 ± 2.89	397.55 ± 3.71	33.98 ± 3.08
Temperature (°C)					
4	585.05 ± 8.25	115.98 ± 6.15	89.24 ± 2.13	199.06 ± 9.29	35.19 ± 1.85
15	1367.2 ± 4.25	239.16 ± 8.14	125.04 ± 1.69	1131.43 ± 2.30	32.58 ± 4.79
Intervals (Days)					
0	195.30 ± 4.26	210.83 ± 2.64	164.14 ± 3.73	24.38 ± 2.21	48.94 ± 1.97
3	672.58 ± 2.54	99.47 ± 6.12	110.58 ± 2.39	457.77 ± 2.14	32.29 ± 1.50
6	895.10 ± 4.27	125.44 ± 4.52	67.19 ± 1.62	724.84 ± 3.13	32.75 ± 3.65
9	1287.33 ± 2.21	234.83 ± 53.15	84.21 ± 5.85	891.25 ± 8.00	30.57 ± 1.07
12	1271.81 ± 9.25	153.40 ± 8.21	97.84 ± 1.00	912.70 ± 3.60	30.20 ± 2.51
15	1533.28 ± 5.26	192.67 ± 1.62	118.52 ± 2.65	980.75 ± 1.24	28.57 ± 1.35
Significance					
Treatments	(*F* = 1115.24) **	(*F* = 35,445.03) **	(*F* = 23,970.39) **	(*F* = 27,561.41) **	(*F* = 18,963.46) **
Colors	(*F* = 284.64) **	(*F* = 5157.02) **	(*F* = 6088.55) **	(*F* = 85,918.17) **	(*F* = 0.019) ^NS^
Temperature (°C)	(*F* = 2489.02) **	(*F* = 8512.48) **	(*F* = 1054.36) **	(*F* = 244,433.7) **	(*F* = 66.97) **
Intervals (days)	(*F* = 500.68) **	(*F* = 1138.20) **	(*F* = 666.34) **	(*F* = 17,773.40) **	(*F* = 421.33) **

**Table 2:** Analysis of antioxidant and phytochemical changes in fresh-cut bell pepper (FCBP) treated or untreated with *Lactobacillus rhamnosus* GG and biochemical agents at different time intervals (days) and preserved in two different temperatures (4 and 15 °C). The results presented the estimated marginal means ± SE. ** significant (*p* < 0.01). TFaC: total flavonoids content, and TPC: total phenolic content.

Sources	DPPH Scavenging (%)	TFaC (OD 450 nm)	TPC (OD 760 nm)
Treatments			
T0	16.95 ± 0.31	0.085 ± 0.018	1.28 ± 0.15
T1	17.37 ± 0.76	0.087 ± 0.005	1.81 ± 0.21
T2	16.16 ± 1.26	0.081 ± 0.007	1.88 ± 0.18
T3	23.27 ± 0.25	0.075 ± 0.010	1.76 ± 0.13
T4	51.75 ± 0.62	0.085 ± 0.004	1.88 ± 0.11
Color of FCBP			
R-FCBP	24.52 ± 2.61	0.084 ± 0.001	1.75 ± 0.17
Y-FCBP	25.69 ± 0.76	0.081 ± 0.010	1.70 ± 0.13
Temperature			
4	24.16 ± 0.37	0.081 ± 0.004	1.71 ± 0.16
15	26.04 ± 0.24	0.085 ± 0.001	1.73 ± 0.11
Intervals			
0	79.95 ± 0.13	0.117 ± 0.013	1.75 ± 0.01
3	17.11 ± 1.41	0.091 ± 0.015	1.86 ± 0.08
6	13.87 ± 0.76	0.091 ± 0.001	1.73 ± 0.09
9	15.16 ± 0.26	0.069 ± 0.02	1.71 ± 0.16
12	10.94 ± 0.77	0.072 ± 0.04	1.63 ± 0.08
15	13.58 ± 1.98	0.056 ± 0.011	1.66 ± 0.02
Significance			
Treatments	(*F* = 16,555.60) **	(*F* = 12.15) **	(*F* = 365.10) **
Color of FCBP	(*F* = 20.24) **	(*F* = 18.07) **	(*F* = 19.48) **
Temperature	(*F* = 52.51) **	(*F* = 11.18) **	(*F* = 8.6) **
Intervals (days)	(*F* = 7175.76) **	(*F* = 7.46) **	(*F* = 30.63) **

**Table 3:** Measurement of physical properties of color and textural changes in fresh-cut bell pepper (FCBP) treated or untreated with *Lactobacillus rhamnosus* GG and biochemical agents at different time intervals (days) and preserved in two different temperatures (4 and 15 °C). *L*: lightness, *a*: red/green value, *b*: blue/yellow value, ∆*E*: color difference. The results presented the estimated marginal means ± SE. ** significant (*p* < 0.01); NS: not significant.

Sources	*L*	*a*	*b*	∆*E*	Texture (g)
Treatments					
T0	22.74 ± 0.20	7.40 ± 1.00	13.76 ± 0.55	8.79 ± 0.20	597.02 ± 5.99
T1	23.36 ± 2.04	9.05 ± 1.38	12.95 ± 1.01	9.32 ± 0.25	693.54 ± 5.49
T2	170.06 ± 2.25	5.80 ± 0.73	18.18 ± 0.51	11.81 ± 0.27	540.99 ± 11.41
T3	27.40 ± 1.99	7.99 ± 0.21	17.56 ± 1.99	8.53 ± 0.69	739.02 ± 2.15
T4	27.96 ± 3.04	7.81 ± 0.17	18.55 ± 2.13	8.20 ± 0.41	449.33 ± 5.88
Color of FCBP					
R	28.92 ± 1.20	17.99 ± 0.18	12.93 ± 0.63	22.46 ± 0.44	659.12 ± 4.87
Y	79.69 ± 1.03	2.24 ± 0.22	19.46 ± 1.52	8.23 ± 1.77	548.84 ± 2.09
Temperature					
4	88.13 ± 2.90	9.01 ± 0.77	18.44 ± 0.55	8.82 ± 1.41	842.41 ± 9.40
15	20.47 ± 1.51	6.22 ± 0.83	13.96 ± 1.73	9.84 ± 1.81	365.55 ± 3.83
Intervals					
0	342.75 ± 0.51	12.46 ± 0.03	26.35 ± 2.69	5.94 ± 0.59	1018.74 ± 8.53
3	240.86 ± 0.72	13.18 ± 0.51	24.95 ± 1.53	6.12 ± 0.44	1054.24 ± 10.35
6	51.73 ± 0.78	7.86 ± 0.26	23.18 ± 0.61	10.93 ± 0.14	708.40 ± 13.20
9	31.71 ± 2.22	8.14 ± 0.66	14.90 ± 0.87	8.59 ± 0.65	524.00 ± 3.61
12	10.63 ± 2.05	3.77 ± 0.84	5.67 ± 1.12	11.79 ± 0.87	252.84 ± 3.34
Significance					
Treatments	(*F* = 3.97) **	(*F* = 122.09) **	(*F* = 53.07) **	(*F* = 5.58) **	(*F* = 83.89) **
Color of FCBP	(*F* = 3.35) **	(*F* = 13.51) **	(*F* = 223.21) **	(*F* = 14.93) **	(*F* = 8.35) **
Temperature	(*F* = 3.35) **	(*F* = 619.08) **	(*F* = 120.36) **	(*F* = 0.11) ^NS^	(*F* = 872.56) **
Intervals (days)	(*F* = 4.95) **	(*F* = 1564.99) **	(*F* = 668.91) **	(*F* = 12.77) **	(*F* = 831.22) **

## Supplementary Materials

The following are available online at https://www.mdpi.com/2304-8158/9/9/1252/s1, Table S1: Microbial counts, such as total bacterial counts (TBC), total fungal count (TFC), total *Salmonella* sp. count (TSC), and total *Listeria* sp. count (TLC), of red fresh-cut bell pepper (R-FCBP) processed with different treatments and preserved at 4 °C and 15 °C. The results were presented as mean ± SE (*n*^−3^). Table S2: Determination of microbial counts of total bacterial (TBC), total fungi (TFC), total *Salmonella* sp. (TSC), and total *Listeria* sp. (TLC) from yellow fresh-cut bell pepper (Y-FCBP) processed with different treatments and preserved at 4 °C and 15 °C. The results were presented as mean ± SE (*n*^−3^).
