# Supplementary material for: Lactobacillus rhamnosus GG and Biochemical Agents Enrich the Shelf Life of Fresh-Cut Bell Pepper (Capsicum annuum L. var. grossum (L.) Sendt)
Source: Foods. 2020 Sep 7;9(9):1252. doi: 10.3390/foods9091252 (PMC7555232; doi:10.3390/foods9091252)
Supplement: Supplementary file 1 [file foods-09-01252-s001.pdf]

SI. Table.1. Microbial counts such as total bacterial counts (TBC), Total fungal counts (TFC), Total *salmonella* sp. counts (TSC), total *listeria* sp. counts (TLC) of Red Fresh cut bell pepper (R-FCBP) processed with different treatments and preserved at 4 °C and 15 °C. The results presented as mean ± SE (n-3).

Factors	Treatments	Temperature (°C)	Storage intervals (Days)/ microbial counts (CFU.g^{-1})					
			0	3	6	9	12	15
TBC (CFU.g^{-1})	T0	4	241.30±2.61	447.04±1.61	660.23±2.87	747.7±3.74	1565.63±4.68	2566.92±6.00
		15	239.10±1.25	1090.23±2.90	2530.36±1.94	3553.60±5.20	4734.55±4.98	4855.23±2.66
	T1	4	4.30±0.97	21.33±2.02	724.99±3.82	1135±2.88	1569.33±2.14	1713.66±6.18
		15	16.16±2.56	1345.75±1.62	1429.26±4.17	2386.49±3.74	2366.25±3.08	2576.15±4.77
	T2	4	156.25±4.50	165.19±2.95	359.42±2.08	535.80±2.74	852.33±12.41	1234±5.85
		15	150.41±3.87	1853.00±12	2643.66±4.09	2143.66±5.36	2136.33±10.98	1155±8.54
	T3	4	154.00±5.85	303.00±3.78	350.25±2.38	465±5	869.64±2.93	1022.31±8.41
		15	156.00±8.52	729.66±4.17	831.66±16.91	1070.66±3.84	1336.66±11.68	1559±14.57
	T4	4	20.66±1.76	175±6.08	228.66±15.67	338±13.65	517.33±4.37	628.34±3.45
		15	20.18±1.31	255.33±5.78	318.08±1.41	522.33±5.78	659.33±5.45	849.33±12.66
TSC (CFU.g^{-1})	T0	4	0.00	1.86±0.33	2.48±0.24	3.29±0.30	4.77±0.13	4.98±0.47
		15	0.00	1.45±0.11	1.89±0.01	2.11±0.03	2.53±0.06	2.87±0.02
	T1	4	0.00	0.00	0.00	0.00	0.00	0.00
		15	0.00	0.00	0.00	0.00	0.00	0.00
	T2	4	1078.10±2.10	72.83±1.38	146.17±2.70	206.46±2.32	344.96±1.26	695.57±1.71
		15	1526.33±4.33	311.33±5.84	910.83±6.43	1162±7.37	1251.77±6.10	1359.31±2.61
	T3	4	0.00	0.00	0.00	0.00	0.00	0.00
		15	0.00	0.00	0.00	0.00	0.00	0.00
	T4	4	82.62±6.90	113.66±5.78	217.46±9.25	191.29±3.70	370.25±4.54	226.40±1.09
		15	89.15±2.48	360.5±2.61	728.67±13.56	2041±28.53	4.43±0.69	14.45±0.62
TLC (CFU.g^{-1})	T0	4	0.00	1.4±0.2	1.16±0.56	1.19±0.07	1.43±0.59	3.25±1.00
		15	0.00	0.88±0.02	1.08±0.03	1.17±0.04	1.79±0.09	2.07±0.05
	T1	4	0.00	0.00	0.00	0.00	0.00	0.00
		15	0.00	0.00	0.00	0.00	0.00	0.00
	T2	4	891.04±10.64	44.40±0.94	152.94±1.38	354.48±2.15	659.22±8.03	747.25±2.51
		15	1069±9.71	411.33±5.81	528.58±8.75	697.03±9.29	1062±6.24	1211±5.50
	T3	4	0.00	0.00	0.00	0.00	0.00	0.00
		15	0.00	0.00	0.00	0.00	0.00	0.00

	T4	4	110.9±4.25	24.2±1.60	82.1±1.62	19.66±0.88	0.00	0.00
		15	112.23±1.48	410±2.88	375±9.53	4.00±1.10	0.00	0.00
TF (CFU.g-1)	T0	4	2.00±0.57	1.66±0.33	67.46±1.23	123.25±1.80	1189±4.61	1322±2.08
		15	4.33±1.35	2481±4.16	2030.33±2.66	2256.66±6.66	2570.66±7.21	2561.33±7.68
	T1	4	23.35±2.74	746.33±2.84	2.66±0.33	172.3±4.12	365.27±7.61	562.66±6.76
		15	26.55±1.15	3518±4.04	2069.5±5.63	2179.5±4.06	2532.33±8.16	2654±17.57
	T2	4	33.35±3.13	178.2±11.80	768.66±10.49	158.9±4.51	1087.33±11.92	1248±3.46
		15	39.32±2.15	654.66±2.60	2358±0.86	2253.31±6.08	2446.5±6	2465±10.01
	T3	4	18.02±1.76	19.59±0.87	25.46±0.66	29.18±0.32	31.27±0.55	36.79±0.95
		15	19.18±1.52	105.28±1.70	180.92±15.45	313.84±4.60	377.79±11.01	429.21±2.54
	T4	4	2.00±0.57	4.98±0.43	57.37±2.62	92.2±3.60	1122.03±1.48	1159.66±21.15
		15	152±17.03	335.36±5.83	1331.16±3.34	2155.33±15.24	2359.33±18.40	2466±10
LAB (CFU.g.-1)	T0	4	0.00	0.00	0.00	0.00	0.00	0.00
		15	0.00	0.00	0.00	0.00	0.00	0.00
	T1	4	0.00	0.00	0.00	0.00	0.00	0.00
		15	0.00	0.00	0.00	0.00	0.00	0.00
	T2	4	0.00	0.00	0.00	0.00	0.00	0.00
		15	0.00	0.00	0.00	0.00	0.00	0.00
	T3	4	119.92±9.06	84.37±2.05	90.35±0.49	78.44±1.64	84.70±1.78	80.38±1.07
		15	126.12±4.15	83.00±1.14	77.38±0.86	71.33±1.19	74.49±0.49	75.35±2.10
	T4	4	127.58±2.90	80.58±1.20	83.69±0.87	77.69±1.07	76.90±0.88	72.45±1.24
		15	115.58±5.26	73.36±2.56	80.88±0.85	64.69±2.42	67.01±0.81	61.58±0.50

SI. Table.2. Determination of microbial counts of total bacterial (TBC), total fungi (TFC), total *salmonella* sp. (TSC), total *listeria* sp. (TLC) from yellow fresh cut bell pepper (Y-FCBP) processed with different treatments and preserved at 4 °C and 15 °C. The results presented as mean ± SE (n-3).

Factors	Treatments	Temperature (°C)	Storage intervals (Days)/ microbial counts (CFU.g^{-1})					
			0	3	6	9	12	15
TBC (CFU.g^{-1})	T0	4	220.24±1.25	472.69±2.89	1109.23±8.03	1833.26±2.00	1937.36±6.74	2932.16±6.54
		15	226.25±2.65	1723.61±2.92	2145.71±5.82	2750.26±7.64	3560.70±1.25	4884.26±5.51
	T1	4	0.00	193.03±6.19	321.99±8.30	534.89±11.48	15.00±0.58	16.33±1.20
		15	0.00	1631.00±3.79	1168.32±7.01	2146.06±9.70	229.00±3.46	334.33±4.63
	T2	4	1014.00±1.85	346.67±6.69	313.98±4.59	425.52±3.09	73.30±1.15	132.33±1.45
		15	1054.00±5.86	1475.67±9.84	1482.37±11.37	3332.67±11.57	836.00±5.86	878.33±4.41
	T3	4	4.67±1.15	14.33±1.20	42.00±5.51	122.28±3.53	156.53±14.22	113.73±2.31
		15	20.67±1.76	562.00±19.97	229.67±8.95	150.67±11.02	165.83±10.54	268.00±14.22
	T4	4	54.00±5.86	208.17±5.95	262.54±0.02	681.45±0.72	804.83±0.42	974.37±2.94
		15	154.00±5.86	439.00±13.01	767.33±11.78	874.67±12.72	1049.27±8.30	1967.53±11.96
TSC (CFU.g^{-1})	T0	4	0.00	0.10±0.01	1.15±0.06	1.56±0.03	1.85±0.04	2.13±0.02
		15	0.00	1.11±0.05	1.38±0.14	1.71±0.08	2.08±0.05	2.66±0.10
	T1	4	0.00	0.00	0.00	0.00	0.00	0.00
		15	0.00	0.00	0.00	0.00	0.00	0.00
	T2	4	742.32±1.25	257.87±1.11	187.06±1.25	233.33±8.33	435.05±1.31	583.15±7.49
		15	757.96±0.91	473.67±2.96	232.06±1.83	614.33±5.21	785.05±9.03	1003.00±4.93
	T3	4	0.00	0.00	0.00	0.00	0.00	0.00
		15	0.00	0.00	0.00	0.00	0.00	0.00
	T4	4	69.25±32.56	33.84±0.87	42.84±1.23	465.42±11.87	6.67±0.88	128.34±1.73
		15	81.80±6.82	457.84±2.68	156.33±0.08	0.00	0.00	8.30±0.42
TLC (CFU.g^{-1})	T0	4	0.00	0.07±0.01	1.03±0.01	1.25±0.15	2.89±0.19	7.78±0.87
		15	0.00	1.32±0.12	1.46±0.06	1.83±0.03	2.15±0.02	2.55±0.06
	T1	4	0.00	0.00	0.00	0.00	0.00	0.00
		15	0.00	0.00	0.00	0.00	0.00	0.00
	T2	4	412.00±2.58	182.33±1.45	81.17±3.94	171.33±2.40	227.50±0.95	396.55±9.39
		15	454.26±5.50	572.67±4.98	26.63±1.27	0.00	0.00	0.00
	T3	4	0.00	0.00	0.00	0.00	0.00	0.00
		15	0.00	0.00	0.00	0.00	0.00	0.00

	T4	4	115.26±1.14	136.00±14.98	85.33±3.53	432.33±10.74	0.00	0.00
		15	118.25±1.73	427.00±7.51	7.33±1.20	0.00	0.00	0.00
TF (CFU.g-1)	T0	4	16.32±2.02	0.73±0.12	4.50±0.17	2.83±0.20	13.44±0.51	35.09±0.72
		15	26.69±4.15	173.00±3.99	3243.33±9.82	3374.00±11.27	1247.00±1.15	1274.50±5.92
	T1	4	15.26±1.12	41.67±1.76	48.65±1.73	5.57±1.13	55.00±1.50	5.00±0.58
		15	16.69±2.23	218.00±7.94	283.00±7.57	1173.83±8.96	622.67±11.89	749.43±1.92
	T2	4	25.26±1.42	8.30±1.35	0.00	0.00	7.83±1.16	47.89±3.91
		15	29.59±3.84	231.67±14.53	1347.67±12.47	2090.33±5.24	1249.67±8.41	1346.33±1.33
	T3	4	19.56±1.72	38.36±1.73	68.58±1.76	108.59±2.02	162.22±13.66	216.26±4.94
		15	18.28±1.78	43.36±1.16	79.59±0.88	112.92±3.93	136.59±2.96	185.34±3.96
	T4	4	0.00	3.77±0.22	208.43±3.54	6.67±0.88	52.99±2.27	74.34±2.14
		15	0.00	344.83±11.55	321.00±15.50	1219.17±18.59	625.23±5.37	776.33±1.33
LAB (CFU.g-1)	T0	4	0.00	0.00	0.00	0.00	0.00	0.00
		15	0.00	0.00	0.00	0.00	0.00	0.00
	T1	4	0.00	0.00	0.00	0.00	0.00	0.00
		15	0.00	0.00	0.00	0.00	0.00	0.00
	T2	4	0.00	0.00	0.00	0.00	0.00	0.00
		15	0.00	0.00	0.00	0.00	0.00	0.00
	T3	4	114.25±1.30	80.92±1.45	85.79±0.62	82.37±0.59	84.56±1.15	80.69±1.23
		15	117.92±2.60	81.39±0.52	79.80±0.78	80.93±1.21	69.57±5.51	67.35±2.67
	T4	4	132.02±1.43	82.46±0.67	79.49±0.39	82.02±0.62	78.49±1.62	71.78±0.40
		15	125.46±3.86	79.82±2.12	77.70±0.91	74.12±1.95	68.38±1.40	61.82±1.84
